# Steady-State Acceptor Fluorescence Anisotropy Imaging under Evanescent Excitation for Visualisation of FRET at the Plasma Membrane

**DOI:** 10.1371/journal.pone.0110695

**Published:** 2014-10-31

**Authors:** Viviane Devauges, Daniel R. Matthews, Justin Aluko, Jakub Nedbal, James A. Levitt, Simon P. Poland, Oana Coban, Gregory Weitsman, James Monypenny, Tony Ng, Simon M. Ameer-Beg

**Affiliations:** 1 Richard Dimbleby Cancer Research Laboratory, Division of Cancer Studies and Randall Division of Cell and Molecular Biophysics, King's College London, London, United Kingdom; 2 Department of Physics, King's College London, London, United Kingdom; 3 UCL Cancer Institute, University College London, London, United Kingdom; The Beatson Institute for Cancer Research, United Kingdom

## Abstract

We present a novel imaging system combining total internal reflection fluorescence (TIRF) microscopy with measurement of steady-state acceptor fluorescence anisotropy in order to perform live cell Förster Resonance Energy Transfer (FRET) imaging at the plasma membrane. We compare directly the imaging performance of fluorescence anisotropy resolved TIRF with epifluorescence illumination. The use of high numerical aperture objective for TIRF required correction for induced depolarization factors. This arrangement enabled visualisation of conformational changes of a Raichu-Cdc42 FRET biosensor by measurement of intramolecular FRET between eGFP and mRFP1. Higher activity of the probe was found at the cell plasma membrane compared to intracellularly. Imaging fluorescence anisotropy in TIRF allowed clear differentiation of the Raichu-Cdc42 biosensor from negative control mutants. Finally, inhibition of Cdc42 was imaged dynamically in live cells, where we show temporal changes of the activity of the Raichu-Cdc42 biosensor.

## Introduction

Many biological processes occur at the cell plasma membrane which constitutes a signalling platform for the cell. For cells in culture, Total internal reflection fluorescence (TIRF) microscopy is the method of choice to image events occurring at the plasma membrane since this technique enables excitation of only those fluorophores that are located very close to the glass coverslip (typically within 100 nm) [1], by generating an evanescent wave at an interface between substrates with refractive index mismatch. This confined excitation provided by the evanescent wave restricts the fluorescence coming from intracellular regions and minimizes the background fluorescence. It thus results in an increase in signal-to-background noise and thus contrast [2], as well as a reduced photodamage in comparison to epifluorescence illumination. It allows visualisation of cellular processes from ensemble measurements down to the single molecule level [3] for a wide range of biological processes such as cytoskeleton dynamics [4], vesicle trafficking [5] and has been successfully combined with super-resolution localization microscopies like PALM [6] or STORM [7] or super-resolution microscopy like TIRF-SIM [8]–[11].

Given its wide-field configuration, which enables fast acquisition rates, TIRF imaging enables observation of dynamic protein interactions at the plasma membrane [12]. TIRF microscopy has already been applied to measurement of protein-protein interactions using Förster resonance energy transfer (FRET) [13], [14], by fluorescence lifetime imaging microscopy (FLIM) [12], [15]–[17] and by acceptor photobleaching [18]–[21]. Fluorescence polarisation constitutes another method of contrast for FRET imaging by measuring the steady-state or time-resolved fluorescence anisotropy with wide field or laser scanning imaging techniques [22]. Monitoring fluorescence anisotropy is the method of choice to follow interactions between identical proteins via homo-FRET, and has been used to investigate protein oligomerisation [23]–[25]. In addition, fluorescence anisotropy can also probe hetero-FRET (i.e. energy transfer between two distinct fluorescent molecules or proteins) [26]. In the presence of an acceptor molecule and where FRET is favourable, donor anisotropy can be observed to increase due to a reduction in the fluorescence lifetime following the Perrin equation [27]. However, the dynamic range of donor polarisation as a result of FRET is limited. Conversely, a low value of anisotropy of acceptor molecules is observed. The highly polarised nature of the donor and the unconstrained orientation of the acceptor molecules results in a highly depolarised acceptor population. Acceptor anisotropy FRET (aaFRET) provides good dynamic range in a system where the fundamental anisotropy (i.e. in the absence of FRET) is high. Fluorescent proteins prove to be particularly good candidates as fluorophores for this technique, since they have rotational correlation times that far exceed their fluorescence lifetime [25], [28]. As the donors undergo minimal rotational diffusion on the timescale of their excited state lifetimes, their emission is predominantly polarised parallel to the excitation light. As such, aaFRET enables the detection of FRET false positives linked to the acceptor direct excitation (common in spectral and intensity based analyses), since every direct excitation of the acceptor is highly polarised and results thus in a measurement of its unperturbed fluorescence anisotropy [26]. This technique is thus an efficient method for measurement of hetero-FRET given its wide dynamic range, fast acquisition rates (<200 ms), and simple and affordable implementation as shown previously [29]–[31]. However, aaFRET requires knowledge or control of the stoichiometry of the FRET pairs and high initial fluorescence anisotropies for both donor and acceptor are required [26], [29]. The constraint on the stoichiometry makes aaFRET an ideal technique to probe intramolecular FRET by monitoring changes of protein conformation in a biological system with a well-known donor: acceptor stoichiometry, intrinsic in FRET biosensor systems.

In this paper, we perform steady-state acceptor fluorescence anisotropy under evanescent excitation to monitor the activity of Cdc42 at the cell plasma membrane using a Raichu-Cdc42 FRET biosensor probe. We show that by measuring aaFRET in a TIRF configuration we could follow dynamically differences in Cdc42 activity by imaging Raichu-Cdc42 FRET biosensors [32], which consist of eGFP as donor and mRFP1 as acceptor [33]. We studied the activity of Cdc42, a member of the Rho GTPase subfamily, which regulates cellular functions such as cytoskeletal reorganization, cell division [34], signal transduction or vesicle trafficking [35]. Cdc42 plays a key role in cell motility as well as invasion and migration [36], [37], which are targeted processes involved in cancer disease [38]–[40]. Furthermore, Cdc42 over-activity has been associated to cancer [41]–[43], immune diseases [44] and neuronal disorders [45]. FRET biosensors have been developed and allow monitoring of the activity of signalling molecules [46], [47] and have helped further our understanding of the signalling pathways involved in oncogenic processes. These single molecule probes permit us to follow the differences in activity of guanine nucleotide exchange factors and GTPases-activating proteins for Cdc42 at the plasma membrane [48]. As a member of the family G proteins, Cdc42 can switch between an active GTP bound state and an inactive GDP bound state. This bipolar behaviour of Cdc42 activity is regulated by three classes of proteins: GEFs (guanine nucleotide exchange factor), GAPs (GTPase activating protein), and GDIs (Guanine nucleotide Dissociation Inhibitor). GEFs promote the exchange of GDP into GTP and thus the conversion of Cdc42 to its active state, which results in the interaction of Cdc42 with its effector proteins and the induction of signalling pathways [49] [50]. GAPs then catalyze the hydrolysis of bound GTP and Cdc42 consequently returns to its inactive state [47]. Conversely, GDIs keeps Cdc42 in its inactive GDP-bound conformation. In order to monitor the exchange between GEFs and GAPs, different FRET probes have been developed [32] and have already helped understanding the signalling pathways that are affecting Cdc42 activity [30], [33], [51]–[55]. When Cdc42 is bound to GDP, the biosensor is in its open form and no FRET can occur between the donor and acceptor. Cdc42 binding to GTP induces intramolecular binding of Cdc42 to p21-activated Kinase (PAK1) which results in the occurrence of FRET [48].

In order to perform live cell FRET imaging of changes of conformation of Raichu-Cdc42 FRET biosensor at the cell plasma membrane, we performed aaFRET with a “through the objective” TIRF configuration [1], which allowed access to the specimen. Evanescent wave excitation and time resolved fluorescence anisotropy measurements were previously employed to study orientation of fluorophores at an interface [56]–[58]. TIRF excitation was achieved using a prism enabling an easy control of the incidence angle, a good spatial separation of the excitation signal and the fluorescence, increasing the signal-to-noise ratio but preventing access to the specimen [59]. Recently, this technique was coupled with donor FRET and permitted probing distances of fluorescently labelled oligonucleotides sequences absorbed on a silica surface, through related conformational changes [60]. Compared to a prism conformation, this “through the objective” TIRF imaging required correction for additional depolarization factors induced by the high numerical aperture [1], [58].

With our TIRF-aaFRET microscope, monitoring conformational changes of the Raichu-Cdc42 biosensor in epifluorescence illumination compared to TIRF excitation elucidated differences in the behaviour of the probe adjacent to and far from the plasma membrane. We compared Raichu-Cdc42 biosensor activity at the plasma membrane to the activity of constitutively inactive mutants and show that TIRF-aaFRET provides superior fidelity of interaction compared to epifluorescence illumination. A dominant negative mutant T17N and an effector mutant Y40C of Raichu-Cdc42 biosensor were thus imaged. T17N mutant is trapped in a GDP-bound state (ie inactive), linked to the replacement of Thr^17^ with Asn, which is known to reduce the affinity of G proteins for guanine nucleotide [61]. For Y40C mutant, the substitution of Cys by Thyr in the effector domain of Cdc42 essential for the binding of Cdc42 to PAK1, inhibits intramolecular binding and consequently reduces the probability of FRET occuring [48].

The Raichu-Cdc42 activity at the cell plasma membrane was finally monitored dynamically upon Cdc42 inhibition with a selective guanine nucleotide binding inhibitor (ML141) and we observed the time-scale of decrease of FRET interaction that is an indirect read-out of Cdc42 activity. These datasets provide a compelling argument for measurement of aaFRET by TIRF illumination due to improvements in the dynamic range of interaction observed on stimulation.

### Theoretical Background: Steady-State Acceptor Fluorescence Anisotropy

Fluorescence anisotropy is a measurement of fluorescence depolarization, and provides information regarding molecular rotation and translational mobility as well as microscopic interactions with other molecules or structures. Depolarization can occur due to fast rotational movement of the fluorophore or non-radiative energy transfer to another fluorophore (via FRET), non-parallel absorption and emission dipole moments of fluorophores. Consequently, fluorescence anisotropy is an accurate tool to monitor protein interactions by the analysis of fluorescence depolarisation. Under continuous linearly polarised illumination, steady-state fluorescence anisotropy *r*, is given by the relation:
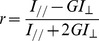
(1)where 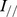
and 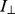
are the fluorescence intensities for polarisations detected parallel and perpendicular to the excitation light polarisation direction (after background subtraction measured). The correction factor, *G*, accounts for differences in the transmission efficiencies of the optics for the two polarizations and is given directly by the ratio of the intensities:



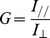
(2)The fluorescence anisotropy is consequently the ratio between the differences of intensities for the two detected polarizations and assuming *G*≈1, the total intensity is directly given by the sum of the parallel and the two perpendicular components relative to the polarisation of the excitation light. When using objective lenses with high numerical aperture (NA) [1], [58], an additional depolarisation factor, related to the ability of high NA objective to resolve oriented fluorophores from a continuous range of angles, is generated [62]–[68]. When collimated beams are focused by high NA objectives, the parallelism of the beams is obviously lost. Consequently, the initial linear polarisation of the beam becomes elliptical linked to the addition of new components. In the case of wide field excitation, depolarisation is only induced at the collection and the objective behaves as an “integrating sphere” resulting in additional projections of the emission dipoles on each of the other two axes relative to the incident excitation [69], [70]. The total intensity must therefore be corrected by a factor *x_NA_* and the total fluorescence anisotropy is calculated accordingly:
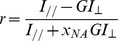
(3)


For objectives with NA≦0.3, no additional depolarisation is induced and *x_NA_* = 2. In the case of objectives with NA>0.3, *x_NA_* decreases [69], [70] and can be determined experimentally [71]. An additional constraint appears when calculating fluorescence anisotropy under evanescent wave excitation in order to determine the initial fluorophore photoselection. At the interface between the sample and the coverslip, for an angle larger than the critical angle for total internal reflection, an evanescent wave is generated. The intensity of this non propagating field decreases exponentially at a rate that depends on the illuminating wavelength, the refractive indexes of the media and the angle of illumination [1]. The penetration depth (<100 nm) allows sub-wavelength confinement and hence localization precision associated with low photobleaching, by ensuring that only fluorophores located very close to the glass-media interface, or in the case of a cell the plasma membrane, are excited. For an evanescent field, in the case of *s*-polarized excitation (perpendicular to the plane of incidence), the polarisation of the evanescent wave generated is perpendicular to the incident plane and thus linearly polarised whereas *p*-polarised excitation induces an elliptically polarized evanescent wave [1], [58], [72]. These characteristics can be used to measure the orientation of fluorophores near optical interfaces by time-resolved fluorescence anisotropy generated for both excitation light polarisations [57], [72]. Excitation light has thus to be *s*-polarised to achieve adequate photoselection [72].

## Materials and Methods

### Plasmids and Reagents

mRFP1-eGFP FRET standards were constructed by the ligation of mRFP1 into the pEGFP-N1 vector, generating random amino acid linkers. The linker for the 7 amino acids (7aa) sequence is RDPPVAT and for the 32aa sequence is KLRILDITSGLETRDASGQSTVPRARDPPVAT. These proteins are termed eGFP-7aa-mRFP1 and eGFP-32aa-mRFP1 respectively and published previously [25].

Raichu FRET biosensors were first developed with CFP/YFP pairs [32] and were obtained from Professor M. Matsuda (Osaka University, Japan). The mRFP1-Raichu-eGFP sensor was synthesized by the excision of the sensor module between YFP and CFP and insertion between the mRFP1 and enhanced GFP (eGFP) in the pEGFP-N1 vector (Clontech) which had been modified by the addition of mRFP1 and the alteration of the multiple cloning sites. The membrane targeting CaaX sequence was cloned after the eGFP, with the resulting construct maintaining the same linkers as the original. The resultant eGFP and mRFP1-tagged Raichu constructs retain the original design of the CFP/YFP- tagged version, i.e., mRFP1-PBD-GTPase-eGFP-CaaX [52]. Upon GTP binding, Cdc42 exhibits a higher affinity toward the Cdc42-interactive binding (CRIB) domain of PAK1 (p21-activated kinase), bringing the two different fluorescent proteins of the biosensor into close proximity and enabling FRET to occur.

Additional negative control constructs derived from the Cdc42-Raichu probe were cloned. In particular, a Thr^17^-to-Asn (T17N) mutation, which is known to have a much-reduced affinity for GTP [73] and a Cys was substituted for Tyr^40^ (Y40C) in the effector domain of Cdc42, which is essential for the binding to PAK1 [74]. Studies using these sensor controls allow us, in principle, to determine the dynamic range of the mRFP1 -Raichu -eGFP.

### Cell lines and sample preparation

The different FRET rulers were expressed in MCF7 breast cancer cells [75] primarily grown in DMEM medium supplemented with 10% foetal bovine serum and 1% penicillin G (100 U/ml)/streptomycin (100 mg/ml) +1% L-glutamine in an atmosphere containing 5% CO_2_/95% air (v/v).

Cells were plated with 70% confluence into multiwell chambers (Imaging Chambers CG, PAA Laboratories) coated with poly-lysine. 24 hours later, cells were transiently transfected with plasmids using a DNA/Fugene6 mixture (Promega, Madison, WI, USA) and were maintained at 37°C in a humidified 5% CO_2_ atmosphere. 24 hours after the transfection, MCF7 cells were fixed with 4% (w/v) paraformaldehyde at room temperature for 15 min. Cells were sodium borohydride treated with 1 mg/mL NaBH_4_ in phosphate buffered saline (PBS) for 5 min and rinsed with PBS before being imaged immersed in PBS.

The different Raichu constructs were transiently expressed in MDA-MB 231 human breast cancer cells [76] primarily grown in DMEM medium supplemented with 10% foetal bovine serum and 1% penicillin G (100 U/ml)/streptomycin (100 mg/ml) +1% L-glutamine in an atmosphere containing 5% CO_2_/95% air (v/v). Cells were plated into multi-well chambers with 70% confluence in the supplemented DMEM medium described above but without the antibiotics. 24 hours later, cells were transiently transfected with plasmids using a DNA/Lipofectamine 2000 mixture (Invitrogen, Carlsbad, CA, USA) and were maintained at 37°C in a humidified 5% CO_2_ atmosphere. 24–48 hours after transfection, cells were imaged live in Optimem supplemented with 25 mM HEPES.

Cdc42 protein inhibition assay was done in HCC1954 human breast carcinoma cell lines [77], primarily grown in RPMI medium supplemented with 10% foetal bovine serum and 1% penicillin G (100 U/ml)/streptomycin (100 mg/ml) +1% L-glutamine in an atmosphere containing 5% CO_2_/95% air (v/v). Cells were plated into multiwell chambers with 70% confluence in supplemented RPMI medium. 24 hours later, cells were transiently transfected with plasmids using a DNA/FugeneHD mixture (Promega, Madison, WI, USA) and were maintained at 37°C in a humidified 5% CO_2_ atmosphere. 24 h after transfection, cells were serum starved for additional 24 hours. Inhibition was performed by using 30 µM of Cdc42 GTPase inhibitor (ML141, Merck Millipore, Merck KGaA, Darmstadt, Germany) in OPTIMEM supplemented with 25 mM HEPES and imaging live cells for up to one hour. Inhibitor stock solutions were made in DMSO at 50 mM.

### Total Internal Reflection Fluorescence Microscope combined with Steady-State Acceptor Fluorescence Anisotropy

**Figure 1:**
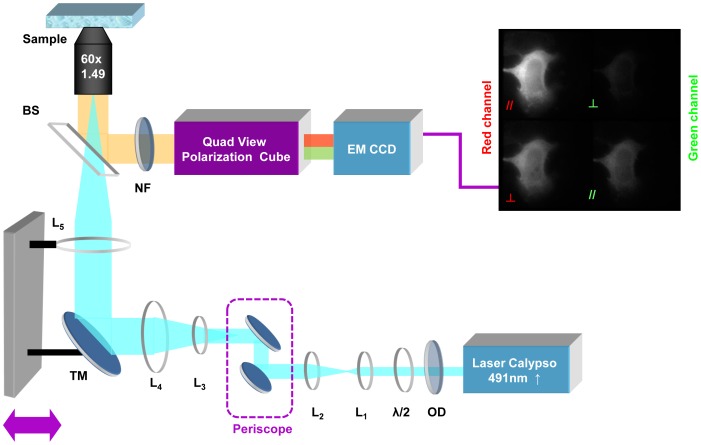
Schematic of the steady-state acceptor fluorescence anisotropy Total Internal Reflection Fluorescence Microscope. OD: Optical density to control the laser power in pupil plane of the objective. λ/2: Half-waveplate in order to change the orientation of the laser output polarisation. L_1_&L_2_: form a telescope to enlarge the beam by a factor of 3. L_3_&L_4_: form a telescope to enlarge the beam by a factor of 5. Beam expansion by a factor of 15 in order to illuminate the entire field of view. TM: Mirror mounted on a translation stage, which enables to switch from an epifluorescence excitation to a TIRF excitation. L_5_: Lens to focus in the back focal plane of the objective (60x, NA = 1.49). BS: Beam splitter. NF: Notch filter to avoid any laser contamination. EMCCD: Electron Multiplying Charge Coupled Device.

Intramolecular FRET in biosensors occurring at, or just below, the plasma membrane was monitored by steady state acceptor fluorescence anisotropy performed on a TIRF microscope. A 491 nm continuous-wave diode-pumped solid-state laser (Cobolt Calypso, Sweden) was used as the excitation source. This laser emitted linearly polarized light (linear, vertical, >100∶1), and a half-wave plate was used to control the orientation of the polarization at the objective back aperture (Fig. 1).

Steady state acceptor fluorescence anisotropy measurements were undertaken for both TIRF and epifluorescence excitation. To perform TIRF, a “through the objective configuration” was chosen in order to have access to the specimen and perform live cell imaging [12], [71]. Experiments were carried out with a Nikon 60× TIRF 1.49 NA objective. To quantify the depolarisation induced by the high NA objective, comparative measurements were undertaken with a low NA objective (Nikon Plan Fluor 4×0.13 NA objective). The lateral position of the focused laser beam in the back focal plane of the TIRF objective was translated with a motorized stage (PT1/M-Z8, Thorlabs UK Ltd), enabling switching from epifluorescence excitation to TIRF with penetration depths ranging between 0 and 300 nm, as previously described [71]. A custom built incubator was implemented on the set-up in order to be able to image at 37°C.

The fluorescence was imaged *via* a polarization-resolved image splitter (QuadView, Photometrics, Tucson, AZ, USA) and relayed on an electron multiplying charge coupled device (EMCCD) (QuantEM 512, Photometrics, Tucson, AZ, USA). The captured fluorescence image was thereby separated into two images with polarization parallel and perpendicular respectively, to the excitation light polarization, for the same region of interest for two different emission wavelength bands of 515±15 nm and 630±35 nm (filters obtained from Chroma Technologies, Rockingham, VT, USA), as described previously [31].

Images containing both spectral and polarization information were acquired using software developed in LabVIEW (National Instruments Ltd, Newbury, UK) with typically 100–200 ms camera exposure time, and electron multiplying (EM) gain ranging between 300 and 400 depending on the sample fluorescence brightness. Laser power was also adjusted depending on the sample fluorescence intensity and was typically between 250 µW and 4 mW measured in the pupil plane of the objective. Consequently, exposure time and laser power were modified to give similar intensities and therefore comparable signal-to noise for each cell/measurement.

### Multiphoton FLIM using TCSPC

Control experiments to quantify FRET efficiency for FRET rulers were performed on a multiphoton, time-correlated single-photon counting (TCSPC) FLIM microscope. A Nikon TE2000E inverted microscope combined with an in-house scanner [55], [78], [79] and Ti: Sapphire laser (Chameleon Vision II, Coherent Inc.) was used for multiphoton excitation of eGFP at 920 nm. All images were acquired at a suitable spatial and time resolution to provide enough photon arrival times to enable fitting of accurate fluorescence decay while avoiding detector “pile-up” and were analyzed by performing a single-exponential pixel fit in TRI2 time-resolved image analysis software [80], [81]. The acquisition speed for single beam scanning was 292 s per image with 3×3 binning (necessary in order to enable fitting of accurate decays) sacrificing also the spatial resolution (resolution of the multiphoton system is diffraction limited but in this instance we acquire data with pixel resolution of 0.61 µm). Temporal accuracy for the FLIM data is determined by the data and associated fitting but generally, measurement error is of the order 50 ps.

### Data Analysis

#### Steady-State Acceptor Fluorescence Anisotropy Calculation

Calculation of the fluorescence anisotropy was performed, as previously described, using custom software in Matlab [31]. Briefly, raw images were split accordingly into the parallel and perpendicular components compared to the excitation polarization for each spectral window. The spatial mapping between the different channels was aligned by imaging fiducial markers (100 nm diameter fluorescent beads) and calculating the affine transform which corrects for lateral shifts and rotations of the particles. An additional transformation calculating the correlation between the different objects in channels was used in order to check the affine function correction.

Before calculating the fluorescence anisotropy using equation (3), a mask was applied on the images in order to select only the region of interest (ROI) and suppress background pixels surrounding the ROI. Thresholds were applied for each spectral window and for each polarisation component in order to subtract the background accordingly. Steady state fluorescence anisotropy was calculated according to equation (3), taking into account *G*, *x_NA_* and after correcting the parallel and perpendicular intensities for bleed-through.

#### Statistical analysis

Statistical analyses were performed using GraphPad Prism software (version 5.00) (GraphPad, San Diego, CA, USA). In order to compare two different populations, we used two-tailed unpaired student *t*-test. If the variances of our two samples were significantly different (*p*-value <0.05), we used a two-tailed unpaired *t*-test with Welch's correction to compare them. Paired *t*-test was used for samples of same cell populations imaged under different conditions.

## Results and Discussion

### Calibration of steady state acceptor fluorescence anisotropy set-up under epifluorescence and TIRF excitation

Calibration of the acceptor steady state fluorescence anisotropy was performed by imaging aqueous Rhodamine B solutions (MW = 479.01 g/mol^−1^, 1 µM, Sigma Aldrich, Saint Louis, MO, USA) with a glycerol content ranging between 0 and 90% (v/v) [71], [82]. The accurate percentage of glycerol per solution was determined by measuring the solution refractive index with a refractometer (Atago, Tokyo, Japan). The viscosity of each solution could then be deduced using calibration tables linking refractive index, percentage of glycerol and viscosity [83]. Each solution was imaged 5 times with 100 ms exposure time with our steady-state fluorescence anisotropy set-up.

Prior to determination of the different correction factors, we accounted for a 6% symmetric polarisation bleed-through in the red channel as previously seen [82]. We corrected for that accurately using equations (4a) and (4b). 
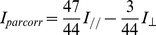
(4a)

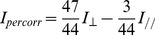
(4b)where 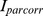
and 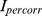
are the fluorescence intensities for polarisations detected parallel and perpendicular to the excitation light polarisation direction, corrected for polarisation leakage and after background subtracted.

The correction factor *G* accounting for the difference of transmission efficiencies of the optics for the two polarisations parallel and perpendicular to the excitation was determined by imaging a non viscous Rhodamine B solution with 0% glycerol content and using equation 2 with the corrected fluorescence intensities given by equations (4a) and (4b). This correction factor was determined with two different objectives 4× Nikon Plan Fluor NA 0.13 and with Nikon 60× TIRF objective NA 1.49 objective. We then measured *G*_4x_ = 1.065±0.003 and *G*_60x_ = 1.075±0.002 (average ±SD).

**Figure 2:**
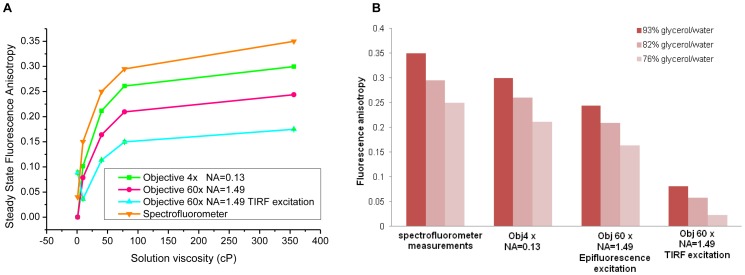
Steady state fluorescence anisotropy of Rhodamine B solutions of different viscosities for objectives with different NA. (A) Variations of the steady state fluorescence anisotropy of solutions of Rhodamine B with different viscosities for objectives with different NA, Objective 4× (NA = 0.13), Objective 60× (NA = 1.49) in epifluorescence and in TIRF excitation without correcting for the depolarisation induced by the high NA objective (*x_NA_* = 2). Fluorescence anisotropy variations with viscosity are shown in (A) and for the different objectives and excitations (B). Each anisotropy value is the average of 5 measurements made on each solution. The average and SD are represented.

The fluorescence anisotropy was then measured for the different range of Rhodamine B solutions using equation (3) combined with (4a) and (4b) for the two objectives without correcting in the first instance for the high NA depolarisation (ie *x_NA_* = 2). As a reference, Rhodamine B steady state fluorescence anisotropy was also measured using a spectrofluorometer (FluoroMax4, Horiba, UK.). As can be seen on Figure 2.A and Table 1, there is a significant decrease in the measured fluorescence anisotropy in the case of solutions imaged with high NA objective which is more pronounced under TIRF excitation. In fact, in evanescent excitation, since light is predominantly collected from fluorophores in close proximity to the glass surface, their emission properties will be modified by the presence of an interface [84]–[86]. Consequently, the closer the dipoles are to the surface, the more energy is transferred into propagating light for angles higher than the critical angle, resulting in redistribution of intensities. Several studies have shown that a high numerical aperture objective and its ability to “observe” an oriented fluorophores from a continuous range of angles reduce the polarisation of the emission [63]–[68], [87]. Corrections have been derived in order to take into account the depolarisation induced by the high NA [63]. However, these formulas are approximations since they assume that no interface is present and it is known to affect the emission polarization behaviour of a fluorophore. A more detailed calculation was thus undertaken which includes near field coupling effect, taking into account that for dipoles close to a surface, some of the near field energy that doesn't propagate is captured by the surface and converted into propagating energy [86], [87]. Thus, the presence of a dielectric surface perturbs the emission of fluorescence in close proximity and is dependent on fluorophore orientation and proximity to surface. In fact, a theoretical study by Hellen *et al.* [87] looked at how a bare glass-water interface affects the angle-dependent intensity, collected power, polarization, and lifetime of the fluorescence emission as a function of the orientation and distance of the fluorophore with respect to the interface, looking at the emission properties of fluorophores considered as fixed power dipole. Another theoretical study done by Burghardt *et al.* [88] by deriving integral expressions for the electric field emitted by an oscillating electric dipole when the dipole is near a dielectric interface, has showed that an interface alters the effective aperture of an objective by reflecting and refracting incident plane waves and it also perturbs the emitted evanescent waves such that some of them become transverse propagating waves with a strong dependence on the distance of the dipole to the interface. More recent works [89] showed that a large part of the radiation of a fluorescing molecule in air or water located in close proximity to a glass surface will be emitted as supercritical angle (SAF) into the glass above the TIR angle. The dependency of the angular radiation pattern of a fluorescing molecule with its distance to the surface can thus be used in order to improve confinement at the detection [90], [91]. Consequently the proximity of the surface is going to affect the emission properties of the dipole and effects are qualitatively similar for both excitation polarization [86]. The presence of the interface and the near field coupling induced under evanescent wave excitation results thus in an increased depolarization effect in TIRF compared to in epifluorescence fluorescence excitation as shown in Figure 2.B. The high NA depolarisation correction factor *x_NA_* was determined experimentally as previously described [71], by considering the measurements made with the low NA objective as reference and extracting the correct *x_NA_* in order to match them with the measurements made with the 60× objective using the following equation:
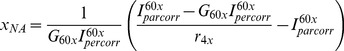
(5)


**Table 1:** Steady-state fluorescence anisotropy of Rhodamine B solutions of different viscosities.

Glycerol (%) (v/v)	Viscosity (cP)	*r_spectro_*	*r_4x_*	*r_60x_^EPI^*	*r_60x_^TIRF^*
0	1.005	0.04	0.000±0.003	0.000±0.003	0.089±0.008
58	9.586	0.15	0.102±0.003	0.07853±0.0002	0.032±0.004
76	40.19	0.25	0.212±0.002	0.1638±0.0005	0.114±0.003
82	77.9	0.295	0.261±0.005	0.210±0.002	0.150±0.006
93	356.2	0.35	0.300±0.002	0.2436±0.001	0.175±0.001

*r_spectro_*: fluorescence anisotropy measured using a spectrofluorometer. *r_4x_*, *r_60x_^EPI^, r_60x_^TIRF^*: fluorescence anisotropy (average ±SD, N = 5), measured with objective 4× (NA = 0.13), objective 60× (NA = 1.49) in epifluorescence and in TIRF excitation respectively, without correcting for high NA depolarisation (ie *x_NA_* = 2). SD: Standard deviation.

In epifluorescence excitation, the *x_NA_* correction factor was determined by correcting measurements made on solutions with glycerol content between 58% and 93% (Table 1) using equation (5). We then measured an averaged *x_NA_* = 1.24±0.03 (average ±SD, N = 20).

In TIRF excitation, *x_NA_* was averaged by correcting measurements made on solutions between 76% to 93% glycerol using values in Table 1 and equation (5) and *x_NA_* ^TIRF^ = 0.45±0.04 (average ±SD, N = 15). The difference in the *x_NA_* correction factors between epifluorescence fluorescence and TIRF excitation thus accounts for the additional depolarisation linked to near field interaction with the surface [85], [86].

### Calibration of aaFRET using FRET rulers in epifluorescence fluorescence excitation

**Figure 3:**
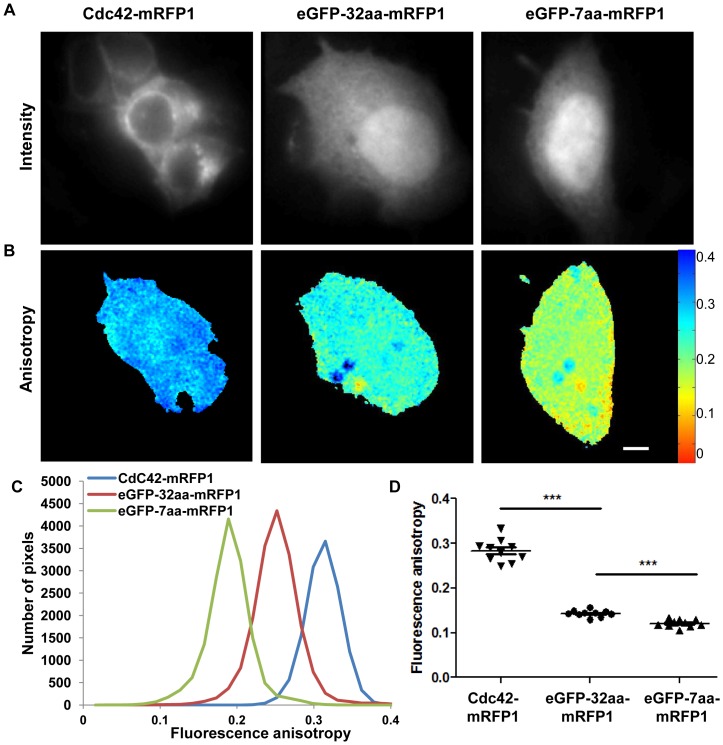
Acceptor fluorescence anisotropy of fixed MCF7 cells transiently expressing eGFP/mRFP1 FRET rulers. Fluorescence intensity (A) and acceptor fluorescence anisotropy maps (B) of fixed MCF7 cells transiently expressing Cdc42-mRFP1 (left side) as a reference, eGFP-32aa-mRFP1 (middle) and eGFP-7aa-mRFP1 (right). Representative histograms of the acceptor fluorescence anisotropy of the corresponding cells (C). Mean values obtained from these histograms for the different cells imaged are then compared using unpaired *t*-test with Welch's correction with 95% confidence intervals (***p<0.001) (D). The scale bar represents 5 µm.

Fixed MCF7 cells expressing eGFP-32aa-mRFP1 and eGFP-7aa-mRFP1 for the FRET rulers and Cdc42-mRFP1 as a reference value for acceptor anisotropy were used to calibrate the aaFRET measurements under epifluorescence fluorescence excitation. The steady-state acceptor fluorescence anisotropy was calculated using equations 3 with equation 4 a&b and total intensity and anisotropy maps were deduced as shown in Figure 3. A & B.

The direct excitation of the acceptor in the case of cells expressing Cdc42-mRFP1 resulted in highly polarized emission (i.e. high anisotropy) (Figure 3.A, left) and a reduction of the fluorescence anisotropy could be seen in the presence of FRET between linked eGFP and mRFP1 (Figure 3.A, middle and right panels). Additionally, the corresponding histograms of the acceptor fluorescence anisotropy (Fig.3.C) underlined a clear decrease of the acceptor fluorescence anisotropy in the case of the different FRET rulers compared to cells expressing only the acceptor (i.e. Cdc42-mRFP1).

Multiphoton, time-correlated single-photon counting (TCSPC) FLIM experiments were also performed to quantify FRET efficiency of the FRET standards using home-build set-up as previously described [79], [92]. FRET efficiencies were calculated using the average fluorescence lifetimes from the donor in absence or in the presence of an acceptor according to equation below [13] and data in Table 2:

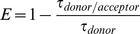
(6)


**Table 2:** Average steady-state fluorescence acceptor anisotropy and fluorescence lifetime of MCF7 cells transiently expressing different eGFP/mRFP1 FRET rulers.

Measurement	Cdc42-mRFP1	eGFP-CaaX	eGFP-32aa-mRFP1	eGFP-7aa-mRFP1
**Acceptor anisotropy (mean±SEM)**	0.284±0.008 (N = 10)	-	0.146±0.005 (N = 9)	0.093±0.002 (N = 7)
**Fluorescence lifetime (mean±SEM) (ns)**	-	2.58±0.04 (N = 9)	2.16±0.01 (N = 9)	1.95±0.02 (N = 9)

SEM: Standard Error of the Mean.

The FRET efficiencies measured were of *E_32aa_* = 0.162±0.006 and E_7aa_ = 0.24±0.01 (average ±SEM, N = 9 for two independent experiments) for eGFP-32aa-mRFP1 and eGFP-7aa-mRFP1, respectively.

The different FRET rulers with respectively 7aa and 32aa linkers could be clearly distinguished (*p*<0.0001,***) using steady state acceptor anisotropy (Table 2 and Fig. 3.D) as well as with FLIM experiments but with a much faster acquisition rate in the case of aaFRET (100 ms compared to 5 minutes acquisitions for FLIM/TCSPC experiments). Given that the fluorescence anisotropy is typically measured on a microscope with an accuracy of 0.01, the different FRET rulers provided a dynamic range from 13 to 20 from the longest to the shortest linkers, whereas FLIM measurements which are typically measured with an accuracy of 0.1 ns offered a dynamic range below 10 (Table 2). Furthermore, it has been shown that even if sensitivity and dynamic range of anisotropy measurements are dependent on signal-to-noise ratio [93], variations in signal-to-noise and level of signal above background can be tolerated, while maintaining the determination of a robust value for the fluorescence anisotropy [30]. In fact, as previously shown [94], large changes in anisotropy can occur for the sensitized emission for modest values of FRET efficiency, which makes it a sensitive technique with high dynamic range to measure interactions between fluorophores. Even so, optimization of imaging conditions (camera exposure time, excitation wavelength and power) ensure the best signal-to-noise ratio is achieved, while minimizing photobleaching and phototoxicity, essential in live cells experiments.

No conversion of the fluorescence anisotropy data was done to FRET efficiency [13] since as shown before [31], it would produce an artificially broad histogram. Rather, we show that our microscope arrangement can be used to distinguish different FRET pairs with a good dynamic range as demonstrated previously [26], [29], in a significantly faster and less expensive manner in comparison to TCSPC-FLIM and without loss in signal-to-noise compared to time-gated system with Rapid Lifetime Determination (RLD) [95]–[97].

### Highlighting differences of activity of Raichu-Cdc42 biosensor at the plasma membrane compared to cytosol

**Figure 4:**
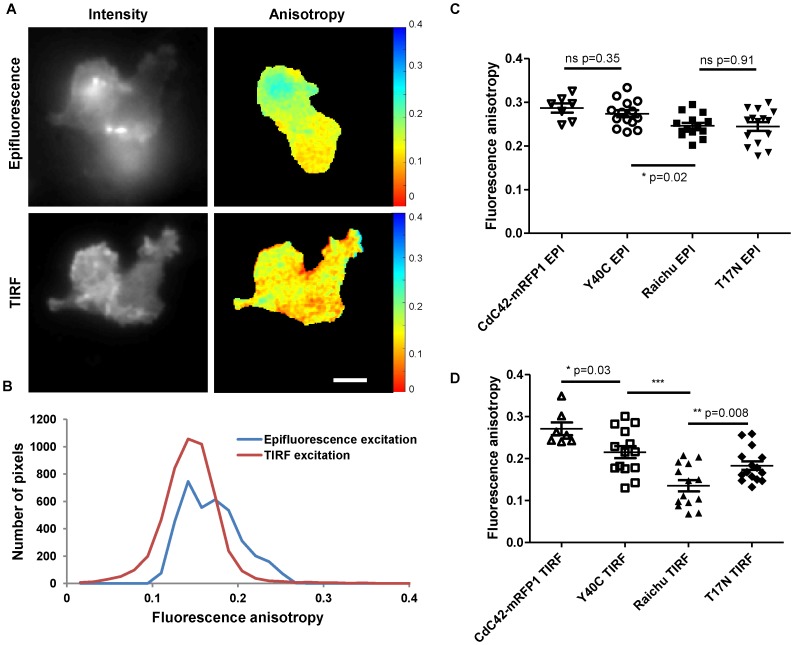
Acceptor fluorescence anisotropy of MDA-MB231 cells expressing Raichu-Cdc42 biosensors imaged in epifluorescence or TIRF excitation. Fluorescence intensity (A left side) and acceptor fluorescence anisotropy maps (A right side) of live MDA-MB 231 cells transiently expressing Raichu-Cdc42 biosensor imaged at 20°C. Representative histograms of the fluorescence acceptor fluorescence anisotropy of the corresponding cells for both excitations (B). Mean values obtained from these histograms on cells expressing different constructs Raichu-Cdc42 biosensor, Y40C mutant, T17N mutant or Cdc42 imaged respectively in epifluorescence excitation (C) and in TIRF excitation (D). These measurements were made on two independent experiments and are compared with a two-tailed unpaired *t*-test with 95% confidence intervals (***p<0.001,**p<0.01, *p<0.05, ns non significant). Transfected MDA-MB 231 cells were typically imaged with 200 ms exposure time for the EMCCD and excitation power between 250 µW to 4 mW depending of the expression level of the construct of interest. The scale bar represents 5 µm.

We monitored Raichu-Cdc42 biosensor activity using aaFRET intracellularly as well as at the plasma membrane of cells by switching from epifluorescence to TIRF excitation. Cells expressing the Raichu-Cdc42 FRET biosensor were imaged live at 20°C and 37°C. First experiments carried out at 20°C showed clear differences in the intensity distribution across the image of the Raichu-Cdc42 construct, linked to the confinement of the excitation in TIRF to the plasma membrane. As can be seen in Figure 4.A in the corresponding intensity images, in epifluorescence excitation, out of focus light is also detected which deteriorates the signal-to-noise and contaminates the signal of interest, whereas in TIRF, the labelling of the whole plasma membrane can be efficiently visualized. In addition to this improvement of the signal visualisation, the average steady-state acceptor fluorescence anisotropy differs with the excitation type. A decrease of the acceptor fluorescence anisotropy was highlighted in TIRF compared to epifluorescence illumination as can be seen in the fluorescence anisotropy maps in Figure 4.A and the corresponding histograms of Raichu-Cdc42 fluorescence anisotropy of the same cell imaged with both excitation types (Fig.4.B). This can be related to a higher activity of the Raichu-Cdc42 biosensor occurring at the plasma membrane compared to intracellularly which correlates with the active GTPase being primarily localized on membranes and Cdc42 involvement with cytoskeleton and actin polymerisation [36], [37]. Additionally, TIRF imaging provides good spatial confinement of the excitation and as a result a better signal-to-noise ratio compared to epifluorescence excitation, where fluorescence signal from planes located above or below the imaging plane are also collected as in Figure 4.A in epifluorescence illumination. This increase in signal-to-noise ratio results in a more symmetric distribution of the anisotropy values in TIRF compared to in epifluorescence excitation (Fig. 4.B).

**Figure 5:**
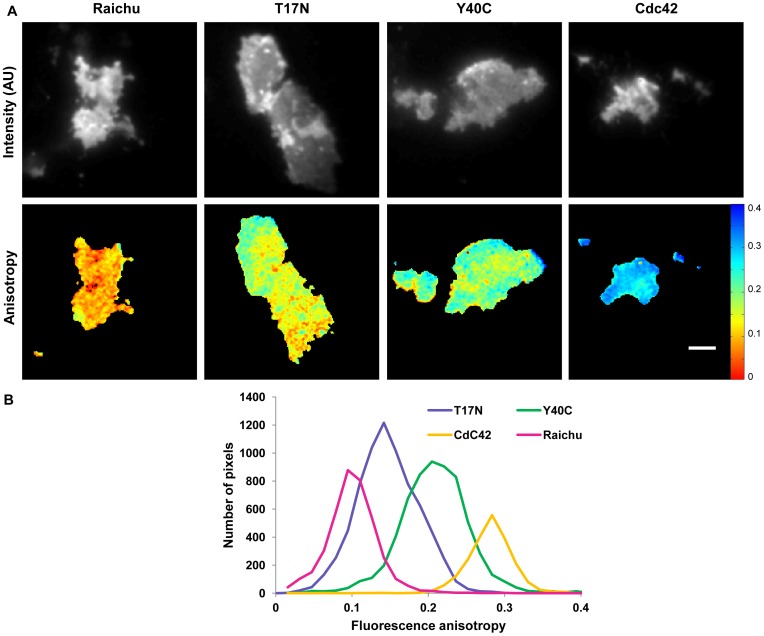
Acceptor fluorescence anisotropy of live MDA-MB 231 cells transiently expressing Raichu-Cdc42 or its different mutants. Fluorescence intensity (A. top) and acceptor fluorescence anisotropy maps (A, bottom) of live MDA-MB 231 cells transiently expressing Raichu-Cdc42 biosensor, T17N mutant, Y40C, mutant or Cdc42 respectively imaged at 20°C. Representative histograms of the fluorescence acceptor fluorescence anisotropy of the corresponding cells (B). The scale bar represents 5 µm.

Having observed significant differences of activity of the Raichu-Cdc42 biosensor depending on excitation condition, we imaged T17N and Y40C mutant biosensors, which constitute negative controls [48]. Cells expressing Cdc42-mRFP1 provided a baseline value for the acceptor fluorescence anisotropy in the absence of donor (i.e. no FRET could occur). Determination of the acceptor fluorescence anisotropy of several cells under epifluorescence illumination for the different constructs and statistical analysis resulted in only a statistically significant difference between Raichu-Cdc42 biosensor and its Y40C mutant (*p* = 0.015,*, Fig. 4.C). In contrast, under TIRF excitation (Fig.4.D), the four different constructs were clearly distinguished with high significance in the case of Raichu-Cdc42 and its Y40C mutant (*p* = 0.0004, ***) and good significance for Raichu-Cdc42 and its T17N mutant (*p* = 0.008,**) as well as Cdc42-mRFP1 and the Y40C mutant (*p* = 0.03,*). Due to the additional spatial information provided by TIRF excitation combined with aaFRET, we were able to clearly distinguish Raichu-Cdc42 and its different mutants and thus to show differences in activity for the different constructs (Table 3). Spatial differences of the fluorescence anisotropy of the different constructs can be seen in the anisotropy maps (Fig.5.A). This result is further supported by the respective distinct histograms of the acceptor anisotropy (Fig.5.B) which showed a significant increase of the acceptor fluorescence anisotropy in the case of theY40C mutant compared to Raichu-Cdc42 biosensor. This confirmed that both Y40C and T17N mutants are negative controls [48], which could not be resolved in epifluorescence excitation linked to both low signal-to-noise ratio (resulting predominantly from fluorescence contamination from out of focus light) and lower observed activity of Raichu-Cdc42 biosensor in the cellular milieu in comparison to at the plasma membrane.

**Table 3:** Average steady-state fluorescence acceptor anisotropy of MDA-MB 231 cells transfected with respectfully Raichu-Cdc42 biosensor, T17N mutant, Y40C mutant and Cdc42-mRFP1 and imaged live at 20°C or 37°C in epifluorescence and in TIRF illumination.

Imaging conditions	20°C		37°C	
Excitation	Epifluorescence	TIRF	Epifluorescence	TIRF
**aaFRET Raichu-Cdc42 (mean±SEM)**	0.246±0.007 (N = 14)	0.14±0.01 (N = 14)	0.172±0.009 (N = 37)	0.135±0.003 (N = 37)
**aaFRET T17N (mean±SEM)**	0.24±0.01 (N = 15)	0.18±0.01 (N = 15)	0.23±0.02 (N = 11)	0.169±0.007 (N = 11)
**aaFRET Y40C (mean±SEM)**	0.274±0.008 (N = 14)	0.22±0.01 (N = 14)	0.227±0.006 (N = 32)	0.237±0.007 (N = 32)
**aaFRET Cdc42 (mean±SEM)**	0.29±0.01 (N = 7)	0.27±0.02 (N = 7)	0.276±0.008 (N = 5)	0.291±0.007 (N = 5)

**Figure 6:**
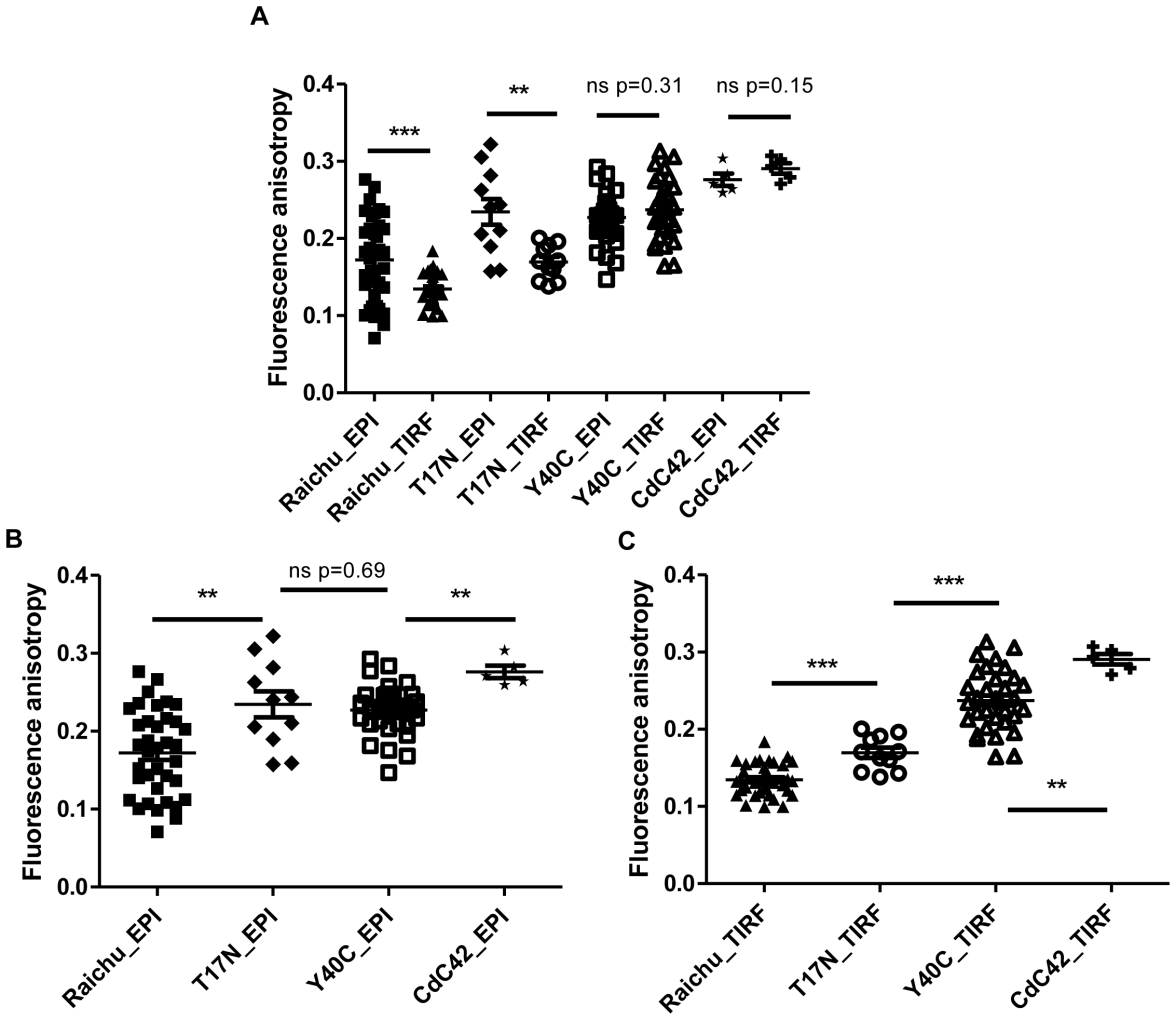
Ensemble steady state acceptor fluorescence anisotropy values of MDA-MB 231 cells expressing different Cdc42 constructs. MDA-MB 231 cells were expressing different Cdc42 constructs and imaged live at 37°C, excited respectively in epifluorescence and in TIRF excitation. Measurements were compared using either two-tailed paired *t*-test (A) or two-tailed unpaired *t*-test with 95% confidence intervals (B, C) (***p<0.001,**p<0.01, *p<0.05, ns non significant).

To monitor Cdc42 activity with changes in temperature, we reproduced the previous experiments at 37°C for the different Cdc42 constructs. Figure 6.A gathers all the data obtained on the different mutants at 37°C and compared the effect of the illumination. It shows that there was a significant decrease of the acceptor fluorescence anisotropy for both Raichu-Cdc42 and its T17N mutant in TIRF excitation compared to epifluorescence excitation which was not the case for the Y40C mutant or the Cdc42-mRFP1 constructs. This is perhaps surprising since T17N mutant is inactive given its GDP-bound state. The Cdc42 component of the sensor has, therefore, a reduced affinity to the PAK1-CRIB binding domain, but not abrogation of binding. As such we must consider the potential energy landscape is sufficiently modified at 37°C to allow significant FRET to occur. Comparing results obtained respectively in epifluorescence illumination (Fig.6.B) and in TIRF (Fig.6.C), we observed higher activity of both Raichu-Cdc42 and T17N at the plasma membrane of the cells compared to intracellularly. Furthermore, in epifluorescence excitation, at 37°C, all constructs were more active since the average acceptor fluorescence anisotropy values were lower compared to those obtained at 20°C (Table 3). Given that no effect of temperature was seen on Cdc42 for both illumination modes (*p* = 0.46 insignificant in epifluorescence, *p* = 0.33 insignificant for TIRF excitation), variations in activity of Cdc42, which must occur due to imaging at physiological temperature, are the most probable cause, although possible phase changes at the plasma membrane [98] and increased probe/effector mobility could also contribute to this effect. This increased activity of the biosensors at 37°C already enabled us to distinguish the different constructs in epifluorescence excitation (although the two negative mutants T17N and Y40C could not be differentiated) (Fig.6.B) and in TIRF excitation, all constructs could be clearly differentiated (Fig. 6.A & C). Use of TIRF excitation for Raichu-Cdc42 constructs and T17N mutant resulted in anisotropy values with a narrow distribution, which is predominantly due to the increase in signal-to-noise ratio in TIRF excitation compared to epifluorescence illumination. The effect of temperature was noticeable on Raichu-Cdc42 and on Y40C in epifluorescence excitation, whereas on the other constructs, no effect of the temperature could be detected for both excitation types. These results reflect the dynamic nature of protein orientation in the biosensor. For the Y40C construct, the binding of Cdc42 to the Cdc42 -interactive binding motif (CRIB) of PAK1 is modified whereas the T17N mutant is trapped in a GDP-bound state (inactive) and the affinity of binding of Cdc42 to PAK1 in presence of guanine nucleotide is reduced but binding of the sensor domain is unaffected. In contrast for Y40C, the binding domain of Cdc42 to PAK1 has been modified preventing binding of Cdc42 and thus the biosensor will always be in an open conformation so FRET is unlikely. Thus, the true negative control upon which we determine the dynamic range of the biosensor must be the Y40C.

### Dynamic inhibition of Raichu-Cdc42 biosensor at the cell plasma membrane

Since Cdc42 activation has been highlighted in various cancer diseases [99], [100], monitoring of its activity could help to clarify the signalling pathways modulated by Cdc42 and design better strategies to target those pathways. Recently a Cdc42 selective guanine nucleotide binding inhibitor (ML141) has been found by high throughput screening [101] and has provided selective, reversible and non-competitive inhibition of Cdc42 [102]. *In vitro* and live cell experiments have shown that ML141 does not prevent guanine nucleotide binding but its association induces the dissociation of the guanine nucleotide resulting in Cdc42 being locked in an inactive conformation. In comparison to other Cdc42 inhibitors like *Clostridium difficile* Toxin B, the association of Cdc42 with membranes should not be inhibited allowing imaging in TIRF excitation.

**Figure 7:**
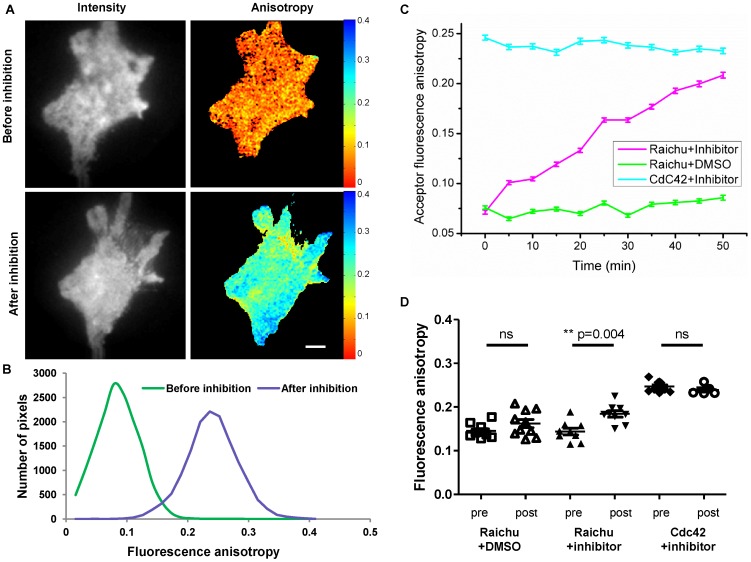
Live inhibition of Cdc42 in HCC1954 cells transiently expressing Raichu-Cdc42 biosensor. Fluorescence intensity (A, left) and acceptor fluorescence anisotropy maps (A, right) of live HCC1954 cells transiently expressing Raichu-Cdc42 biosensor before (A. top) and after (A. bottom) addition of Cdc42 inhibitor. The scale bar represents 5 µm. (B) Corresponding representative histograms of the fluorescence acceptor anisotropy before (A top right) and after inhibition (A bottom right) are shown. (C) Time lapse of acceptor fluorescence anisotropy (±SD) after 30 µM addition of Cdc42 inhibitor for cell expressing respectively Raichu-Cdc42 biosensor (pink), Cdc42 (blue) or after addition of 30 µM DMSO for cell expressing Raichu-Cdc42 biosensor (green). Images were taken every 5 minutes (200 ms exposure for EMCCD) for 50 minutes. (D) Ensemble steady state acceptor fluorescence anisotropy values obtained on HCC1954 cells expressing Raichu-Cdc42biosensor before (pre) and after (post) addition of Cdc42 inhibitor, or before and after addition of DMSO, or for cells expressing Cdc42 before and after addition of Cdc42 inhibitor. Cells were imaged live at 37°C, excited in TIRF excitation.

Cdc42 inhibition was achieved on Raichu-Cdc42 biosensor expressed in HCC1954 cells and imaged live at 37°C, before and after Cdc42 GTPase inhibitor addition (Fig.7.A&B). As can be seen on the fluorescence anisotropy maps pre- and post-addition of the inhibitor (Fig. 7.A) and the corresponding histograms (Fig.7.B), a clear increase of the acceptor fluorescence anisotropy of the Raichu-Cdc42 biosensor was measured 50 minutes after addition of the inhibitor. Furthermore, time-lapse experiments acquiring images every 5 minutes to dynamically monitor Cdc42 inhibition at the cell plasma membrane were undertaken. From these time lapse data, both total intensity and the corresponding acceptor fluorescence anisotropy maps were extracted (Movies S1&S2). The average fluorescence anisotropy extracted from this time lapse highlights a clear increase of acceptor fluorescence anisotropy after Cdc42 inhibition (Fig.7.C). These measurements were repeated in 10 cells and the anisotropy measured before and after inhibition showed a significant increase of the fluorescence anisotropy after inhibition (Table 4 and Fig.7.D, *p* = 0.004, **). In order to confirm that the increase in fluorescence anisotropy was directly related to Cdc42 inhibition, control measurements were undertaken by imaging Raichu-Cdc42 biosensor in 30 µM DMSO in order to ensure that the effect on the fluorescence anisotropy was linked to the inhibitor and not to the carrier. Measurements of the fluorescence anisotropy before and after addition of the inhibitor for several cells (Fig.7.D), and time-lapse experiments (Fig. 7.C) indicate a stable and high activity of the biosensor over the imaging period, without phototoxicity/photodamage induced by the illumination or effect of the carrier. The reference value for the open conformation (i.e. non interacting form) was given by imaging cells expressing Cdc42 –mRFP1 in presence of the inhibitor. In that case too, no effect of the inhibitor was observed either on time lapse (Fig.7.C) or on ensemble measurements (Table 4 and Fig.7.D). As seen in Figure 7.C, after 50 minutes of treatment with the inhibitor, the fluorescence anisotropy of the Raichu-Cdc42 biosensor went from low anisotropy values (comparable to when imaged in DMSO) to reach the fluorescence anisotropy of cells expressing Cdc42-mRFP1 (i.e. baseline value for absence of FRET). Thus, the increase in the acceptor fluorescence anisotropy is directly related to a decrease in the FRET efficiency and consequently to a change of conformation of the Raichu-Cdc42 biosensor from an active (GTP-bound) conformation to an inactive (GDP-bound) conformation. We noticed that the response to inhibition was cell specific in terms of efficiency and time to response after inhibitor addition. On average, the fluorescence anisotropy was increased by 25% after 50 minutes treatment (Table 4). This imaging time window seemed to be optimal in order to have an effective inhibition of Cdc42 activity without inducing phototoxicity/photodamage. In addition to a clear decrease of the intramolecular FRET of the Raichu-Cdc42 biosensor (Fig.7.C), intensity images also showed morphological changes of the cell shape, which might be linked to the induced inhibition of Cdc42 related filopodia formation and cell migration (Movie S1).

**Table 4:** Average steady-state acceptor fluorescence anisotropy before and after Cdc42 inhibition of HCC1954 cells transiently expressing different Raichu-Cdc42 biosensors.

Average acceptor anisotropy	Raichu +DMSO	Raichu +Inhibitor	Cdc42 +Inhibitor
**Before inhibition**	0.145±0.005 (N = 10)	0.144±0.008 (N = 9)	0.247±0.003 (N = 11)
**After inhibition**	0.162±0.009 (N = 10)	0.184±0.007 (N = 9)	0.239±0.005 (N = 9)
***p*** **-value**	0.124 ns Unpaired t-test	0.004 Paired t-test	0.220 ns Unpaired t-test

### Conclusions

We combined TIRF Microscopy with steady-state acceptor fluorescence anisotropy imaging (aaFRET) and observed intramolecular FRET under evanescent wave excitation. Correction factors accounting for the additional depolarization factors induced when imaging in TIRF with a high NA objective were determined by calibration measurements made on Rhodamine B solutions of different viscosities with those made with a low NA objective. This technique enabled us to distinguish different eGFP/mRFP1 FRET rulers with significant gain of speed and cost compared to fluorescence lifetime imaging microscopy.

We then highlighted differences of activity of the FRET Raichu-Cdc42 biosensor with a higher activity measured at the cell plasma membrane compared to intracellularly. Furthermore, imaging in TIRF provided better conditions in terms of signal-to-noise ratio which helped differentiate Raichu-Cdc42 biosensor from its different negative mutants T17N and Y40C. Live inhibition of Cdc42 in the Raichu-Cdc42 biosensor was demonstrated by following the decrease in the related intramolecular FRET indicating changes of conformations of the probe at the cell plasma membrane. We consequently demonstrated intramolecular FRET imaging in wide-field configuration in TIRF excitation with a good contrast and dynamic range as well as fast acquisition rates. The additional spatial information provided insights about Cdc42 activation and underlined its clear inhibition. Furthermore, since Cdc42 has been associated with a migratory behaviour which seems to be dependent on the cell lines as recent studies have shown [40], this new technique could, given its fast acquisition rates, provide quantitative method to test the activation of Cdc42 and to associate it to a migratory or anti-migratory role depending on the cancer cell line.

Whilst FRET biosensors constitute ideal systems to use with aaFRET given their one-to-one stoichiometry, this technique could be extended to imaging protein-protein interactions as shown before [31]. An extra calibration is then required prior to imaging in order to determine the different concentrations of the free donor, free acceptor and of the FRET pairs [31], [103].

## Supporting Information

Movie S1 **Fluorescence intensity time lapse video of HCC1954 cells transiently expressing Raichu-Cdc42 biosensor and under Cdc42 inhibition.** Cells were imaged live before (time 0) and after addition of Cdc42 inhibitor, every 5 minutes (200 ms exposure for EMCCD) for 60 minutes.
(AVI)

Movie S2 **Fluorescence anisotropy time lapse video of HCC1954 cells transiently expressing Raichu-Cdc42 biosensor and under Cdc42 inhibition.** Cells were imaged live before (time 0) and after addition of Cdc42 inhibitor, every 5 minutes (200 ms exposure for EMCCD) for 60 minutes.
(AVI)
